# Medical Students’ Acquaintance with Core Concepts, Institutions and Guidelines on Good Scientific Practice: A Pre- and Post-questionnaire Survey

**DOI:** 10.1007/s11948-020-00215-3

**Published:** 2020-04-15

**Authors:** Katharina Fuerholzer, Maximilian Schochow, Richard Peter, Florian Steger

**Affiliations:** Institute of the History, Philosophy and Ethics of Medicine, Ulm University, Parkstraße 11, 89073 Ulm, Germany

**Keywords:** Ethics, Germany, Medical education, Medicine, Questionnaire, Scientific integrity

## Abstract

German medical students are not sufficiently introduced to the ethical principles and pitfalls of scientific work. Therefore, a compulsory course on good scientific practice (GSP) has been developed and implemented into the curriculum of medical students, with the goal to foster scientific integrity and prevent scientific misconduct. Students’ knowledge and attitudes towards GSP were evaluated by a pre-post-teaching questionnaire survey (n = 239). Most participants initially had startling knowledge gaps in the field. Moreover, they were not acquainted with core institutions on GSP, the office of ombudsperson and the nationally binding guidelines on GSP. The pre-post-teaching comparison showed statistically significant improvement in all areas tested; moreover, after the course participants confided more trust in GSP institutions. Applying ethical rules into practice can be challenging; therefore, students need to learn to work independently with guidelines on GSP and should be introduced to institutions providing further guidance. As our study has shown, students are very willing to pursue a scientific career based on integrity and honesty, however, they lack the knowledge how to do so. In light of our results, we therefore recommend to integrate courses on GSP already at an early time into the mandatory curriculum of medical students.

## Introduction

In 1997, Germany was shaken by a research scandal: Between 1988 and 1996 cancer researcher Friedhelm Herrmann from Ulm University and laboratory head Marion Brach had both forged own data and claimed ideas and results of others as their own (König 1997). In reaction to this scandal, the German Research Foundation (Deutsche Forschungsgemeinschaft, DFG), Germany’s central self-governing organization for research, appointed a taskforce with the mandate to (1) explore causes of dishonesty in sciences, (2) discuss preventive measures, and (3) make recommendations on how to safeguard mechanisms of professional self-regulation in sciences. The results were published in 1998 in the recommendation “Safeguarding Good Scientific Practice,” which was reissued in an updated version in 2013 (German Research Foundation 2013). A revised code of conduct was published in 2019, which now forms the country’s core guideline on good scientific practice (GSP) and is binding for all scientists in Germany (German Research Foundation 2019a).

Twenty years after the Herman and Brach scandal, a lot has been done to improve the situation, thanks to the joint efforts of core scientific institutions in Germany such as the German Research Foundation, the German Rectors’ Conference, and the Max Planck Society. To foster scientific integrity and prevent scientific misconduct already at an early stage of medical training, the teaching project “Safeguarding Good Scientific Practice in the Curriculum of the History, Philosophy and Ethics of Medicine” was started at Ulm University in 2017, with the aim to implement courses on GSP into the mandatory curriculum of medical students. To the best of our knowledge, this is the first time that this has been done at a German university. The main objectives of the project were threefold: (1) impart knowledge on core concepts, institutions and guidelines on GSP, (2) train students’ ability to identify poor research behaviour and know how to react on it, and (3) build an attitude that fosters scientific integrity and prevents scientific misconduct. The course was assessed in form of a pre-post-teaching questionnaire. Both the course and the questionnaire survey were tested in a pilot phase.

We have already published the project’s concept, teaching units and materials (Fuerholzer et al. 2019). In addition to the previous paper, this article is focused on the results of the questionnaire survey. In particular, we will first delineate students’ pre versus post-teaching knowledge and attitudes towards central concepts, institutions and guidelines on GSP. Building up on this, we will then discuss the necessity to (1) train students’ ability to recognize poor research behaviour and identify it as ethically problematic by combining practical case vignettes with theoretical information, (2) foster their trust-based acquaintance with GSP institutions, in particular with regard to ombudspersons, and (3) implement courses on GSP into the mandatory curriculum of medical students.

## Materials and Methods

We developed teaching units and materials for a 180-min compulsory course addressed at medical students in their 3rd year, with the aim to foster scientific integrity and prevent scientific misconduct from the very start of their scientific training (Fuerholzer et al. 2019). The overall aim of the course was to familiarize students with abstract norms of GSP (“scientific integrity,” “scientific misbehavior,” “scientific self-control”), to sensitize them for scientific realms where adhering to said norms is of particular importance (“scientific publishing,” “intellectual property,” “empirical data”), and to familiarize them with central institutions and guidelines they can consult in case they fear to perpetrate the principles of GSP or observe poor research conduct in others (Table 1). For the course, we worked with the student-centered teaching method of problem-based learning (PBL), an established method that puts a specific case or scenario in the foreground (Albanese and Mitchell 1993; Schmidt et al. 2011; Wood 2003; Jones et al. 2010; Bosch-Barrera et al. 2015; Nolte et al. 2015; Fuerholzer et al. 2019). Students were taught both in form of theory-based lectures and practice-based case discussion rounds (Table 1). During case discussions, students worked with hypothetical case vignettes and with excerpts from central guidelines on GSP (German Research Foundation 2013; Ulm University 2009).

**Table 1:** Syllabus of GSP sessions “GSP: part 1” and “GSP: part 2”: structure and topics

	Structure	Topics
GSP: part 1 (90 min)	Introduction	Historical background and status quo of GSP in Germany
		Institutions and guidelines on GSP in Germany
		Learning objectives of “GSP: part 1”
	Case Discussion 1: “Scientific Integrity”	Definitions and dimensions of “good scientific practice,” “scientific integrity,” “scientific misbehavior”
		Finding and understanding central guidelines on GSP
	Case Discussion 2: “Scientific Publishing”	Scientific articles
		Ethical aspects (overall goals of scientific publishing, publishing negative results)
		Practical aspects (structure, content, form, addresses)
		Scientific authorship
		Ethical and legal aspects of scientific authorship (criteria, co-authorship, order of authors)
		Preventing scientific misbehavior in the context of scientific authorship (honorary authorship, ghostwriting)
GSP: part 2 (90 min)	Introduction	Brief recap of “GSP: part 1”
		Learning objectives of “GSP: part 2”
	Case Discussion 1: “Scientific Self-control”	Definitions and dimensions of “scientific self-control”
		Preventing scientific misbehavior in the context of sciences (ombudspersons, ethics committees, whistleblowing, ethical and legal aspects of scientific misbehavior)
	Case Discussion 2: “Intellectual Property”	Definitions and dimensions of “intellectual property”
		Preventing scientific misbehavior in the context of intellectual property (ethical and legal aspects of plagiarism and self-plagiarism)
	Case Discussion 3: “Empirical Data”	Definitions and dimensions of “empirical data”
		Preventing scientific misbehavior in the context of empirical data (fabrication, manipulation, and falsification of empirical data)

The course consisted of 2 consecutive sessions that each lasted 90 min (total duration of course: 180 min). The time span between the sessions was 1–4 weeks. To compare the impact of the course, students’ knowledge and attitudes on GSP were evaluated in form of a pre-post-teaching questionnaire, which was administered at the beginning of the first session, and at the end of the second session. The post-teaching questionnaire was identical with the pre-teaching questionnaire and omitted sociodemographic items.

The questionnaire covered three main areas: First, we collected sociodemographic data and assessed whether the students had already been confronted with issues of scientific work in general and GSP in particular. Second, we assessed students’ knowledge and attitude towards scientific misconduct. In this section, students were given three fictitious case vignettes that each addressed a typical form of scientific misconduct, for example plagiarism or data manipulation (“Appendix” section). In each vignette, we explored students’

- knowledge: 4 items, “yes/no,”
- attitude: 4 items, 5-point Likert-scale ranging from “strongly agree” to “strongly disagree” with additional “prefer not to answer” option,
- previous contact with the specific topic of the case vignette: 1 item, “yes/no.”

Third, we examined participants’ familiarity with core institutions and guidelines on GSP.

Students were given information on the background and purpose of the teaching project and the accompanying evaluation both in written and in verbal form; moreover, they were informed that participation was voluntary and anonymous. Data normality was checked using the D’Agostino-Pearson omnibus test. Paired categorical data were compared using McNemar test. Since continuous data was not distributed normally, Wilcoxon signed-rank test was used. A significance level of *p* < 0.05 was applied in all analyses.

## Results

### Study Population

During the implementation of the project, a total of 323 students were registered for the course. Of these, 301 (93.2%) participated in the first session, and 281 (87.0%) participated in the second session (missing data: absences due to illness etc.). 239 (74.0%) students took part in both the pre- and post-evaluation (missing data: decision not to participate etc.). The questionnaires of students with complete data were included in the analysis and are presented in this paper (n = 239). 148 (61.9%) participants were female, 89 (37.2%) male, 1 (0.4%) participant was intersexual, 1 (0.4%) did not state the sex. These data are representative for German medical students in general (Destatis 2019). The mean age was 23.1 years (SD = 4.2). The vast majority of the participants were in their 3rd year (5th and 6th semester) of medical training (n = 229, 96.2%) (deviating data: students who had to repeat the course etc.).

### Students’ Background Knowledge and Attitude Towards GSP

An aim of the survey was to evaluate students’ background knowledge on GSP. In the pre-teaching questionnaire, the majority of students explained that the course constituted their first contact with GSP: A mere 43 (18.0%) stated that issues of GSP had been part of their studies so far. Moreover, prior to the course only 45 (18.8%) declared to have already attended an event that dealt with issues of GSP. Only a minority had already gathered practical experiences with scientific work (e.g. as a research assistant or doctoral student) (n = 64, 26.8%), and expressed interest in pursueing a future career in sciences (n = 40, 16.7%).

Against this backdrop, it will come as no surprise that both the questionnaires and class discussions revealed knowledge gaps in almost all areas covered, in particular with regard to issues of authorship and intellectual property. The comparison of students’ answers pre versus post-teaching yielded statistically significant improvements in this regard (“Appendix” section). One of the main changes was found with regard to issues of intellectual property: While prior to the course, ¼ of students showed difficulties in recognizing forms of plagiarism (wrong answers: 24.5%), after the course, almost all students answered these questions correctly (wrong answer: 3.4%) (*p* < 0.0001). Next to that, we also wanted to learn about students attitudes towards poor research behaviour. The post-teaching questionnaire indicated an increased sensitivity in this regard. This can again be seen by the example of intellectual property, as after the course the majority of participants regarded plagiarism as ethically problematical or very problematical (n = 30, 12.6% and n = 201, 84.1%, respectively). Despite their initially low level of knowledge, the vast majority of students rendered GSP as important (n = 90, 38.5%) or very important (n = 51, 21.8%). These numbers increased even more after the end of the course (“important”: n = 102, 43.6%, “very important”: n = 101, 43.2%) (*p* < 0.0001).

### Students’ Acquaintance with Institutions and Guidelines on GSP

**Fig. 1:**
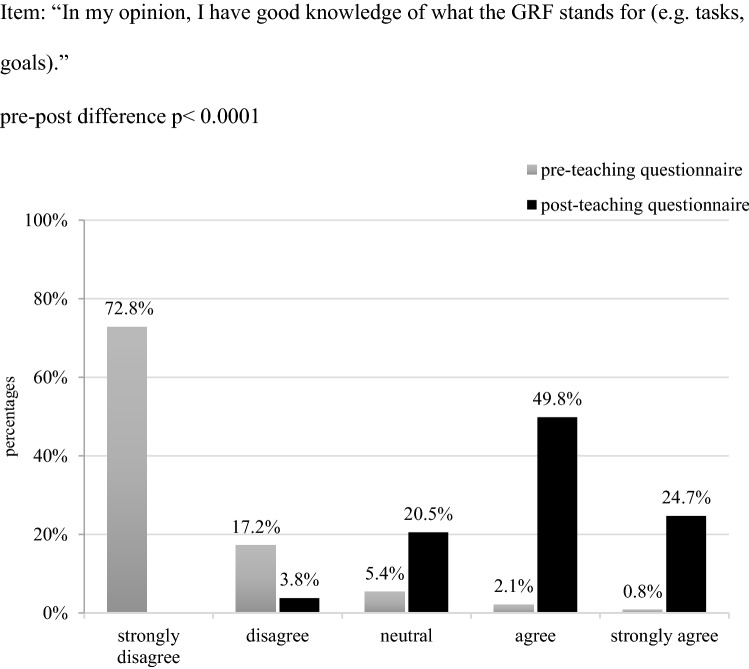
Students’ self-assessed familiarity with the German research foundation in percentage (n = 239)

We were interested in whether students had already heard of the central institutions and guidelines on GSP in Germany. Prior to the course, most student responses revealed alarming knowledge gaps: Not even half (n = 102, 42.7%) of the participants had heard of the German Research Foundation before the course. It thus came as no surprise, that when asked whether they felt to have good knowledge of the tasks and goals of this institution, the majority of students answered in the negative. This improved significantly by the end of the course (*p* < 0.0001) (Fig. 1).

**Fig. 2:**
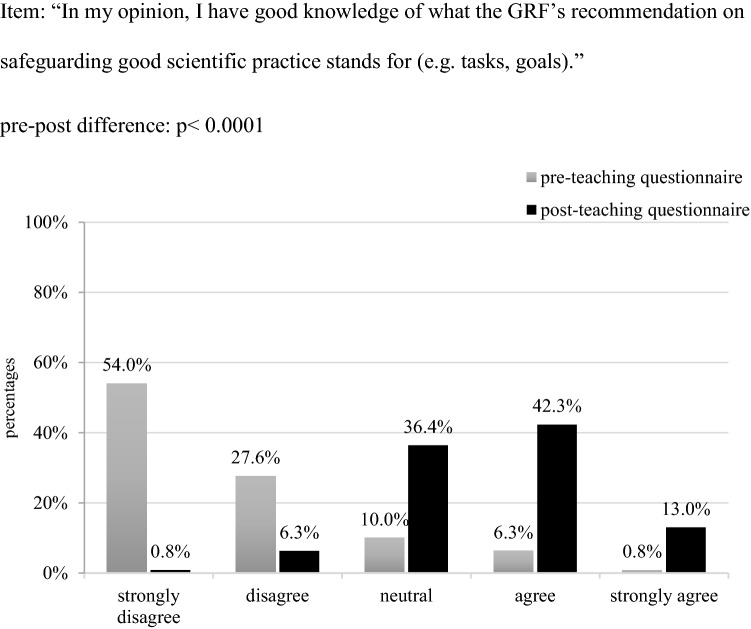
Students’ self-assessed familiarity with the German research foundation’s recommendation on GSP in percentage (n = 239)

With regard to the German Research Foundation’s nationally binding recommendation “Safeguarding Good Scientific Practice,” before the course only 2 (0.8%) participants explained to have read this text in part or in whole. Consequently, when asked about their familiarity with the tasks and goals of this text, the vast majority of participants stated to have no good knowledge of this guideline (Fig. 2). As expected, the pre-post-teaching comparison revealed a statistically significant improvement in this regard: After the course the majority of participants felt to have better knowledge of the tasks, goals etc. of both the German Research Foundation and its recommendation (*p* < 0.0001) (Figs. 1, 2).

### Students’ Acquaintance and Trust in Ombudspersons

**Fig. 3:**
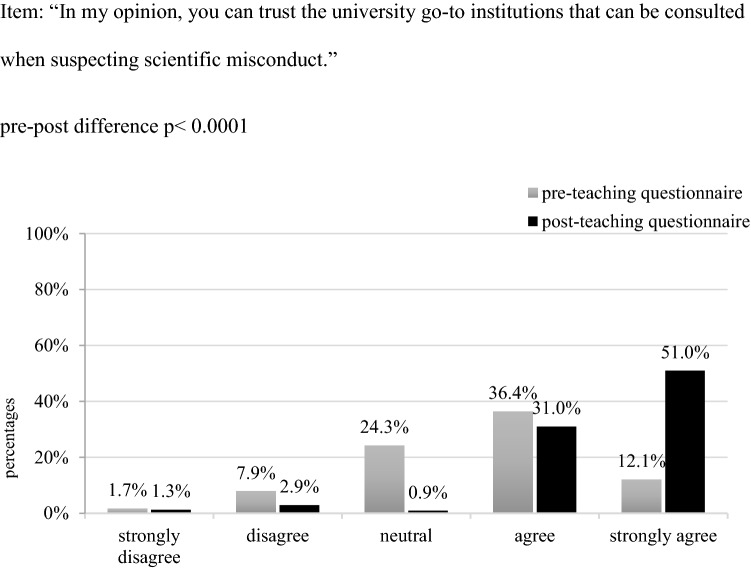
Students’ trust in university institutions on GSP in percentage (n = 239)

Next to that, we assessed students’ acquaintance with institutions they can consult on issues of GSP. Prior to the course, most participants said to have never heard of the office of ombudsperson (n = 222, 92.9%); none of them had previous contact with this institution (e.g. made use of its service or attended an event) (n = 238, 99.6%). Students’ acquaintance improved significantly after the course: When asked again, the majority of students felt to have good (n = 75, 31.4%) or very good (n = 74, 30.8%) knowledge of the tasks of an ombudsperson (*p* < 0.0001). In addition, we assessed students’ trust in university institutions they can turn to in case of a suspected scientific misconduct. Statistical analyses showed that already before the course, almost 50% of students expressed their trust in said institutions. This increased noticeably during courses: When asked again at the end of the second session, almost 80% confided trust or even strong trust (*p* < 0.0001) (Fig. 3).

## Discussion

### Combining Theory and Practice

As the pre-teaching questionnaire showed, most students’ were not familiar with the core institutions and guidelines on GSP (Figs. 1, 2). Next to that, the majority had not been exposed to issues of GSP neither in their previous studies nor any university or non-university event, or had gained any scientific work experience so far. As this data suggests, the majority of students are thus at risk to start their scientific career without being sufficiently prepared neither through theory nor practice for the ethical requirements of scientific work. Students’ initial difficulties to recognize poor research conduct and identify it as ethically problematic can be seen as a reflection on this alarming situation.

Against this backdrop, it seems vital to familiarize students with the core concepts, institutions and guidelines on GSP. It goes without saying that a short-time course can provide students only with a basic understanding of GSP. For that reason, we encouraged students’ to independently deepen and broaden the gained knowledge after the end of the course. To help them fulfil this task, we designed teaching methods and materials that help to foster students’ self-learning competences. In this context, the student-centred teaching method of problem-based learning (PBL) can facilitate practical access to the complex theories and concepts of GSP even for students with no prior experience to scientific work: PBL is an established method that puts a specific case or scenario in the foreground; by analysing and discussing the case and given questions and/or tasks in small groups, students can thus train their abilities of critical thinking, problem solving and self-directed learning (Albanese and Mitchell 1993; Wood 2003; Jones et al. 2010; Bosch-Barrera et al. 2015; Nolte et al. 2015; Fuerholzer et al. 2019). When it comes to GSP, it seems crucial to provide students both with practical real-life scenarios and theoretical information. This need was confirmed during class discussions, as students found guidelines on GSP ambiguous und difficult to understand. Moreover, they were insecure how theoretical, abstract norms like “integrity” can be applied into practice, which is hardly surprising in view of their lack of practical experiences and theoretical preknowledge. We tackled this challenge by providing students with a mixture of theoretical information, readily accessible, practical case vignettes, and excerpts from central guidelines out of scientific practice (Ulm University 2009; German Research Foundation 2013). Students then worked independently in small groups, before discussing their results with the rest of the class.

Combining theoretical texts with practical case discussions cannot only be seen as a fruitful way to train students’ reading comprehension, and to familiarize them with the specific modes of thinking, reasoning and wording of such guidelines, but also as a means to enhance their understanding of correlations between theory and practice. The post-teaching data indicates the suitability of this approach, considering students’ significantly improved ability to recognize examples of poor research conduct and identify them as ethically problematic—skills that are indispensable presuppositions for students’ faculty to apply abstract rules into practice.

### Fostering Trust-Based Acquaintance with GSP Institutions

Unfortunately, the alarming data of our pre-teaching questionnaire seems quite representative for the overall situation in Germany: According to previous findings, many doctoral students have startling knowledge gaps regarding basic concepts of GSP (Gommel et al. 2015; Nolte et al. 2015; Can et al. 2016; Fuerholzer et al. 2019), or have even been involved in severe scientific misconduct (Gommel et al. 2015). What is worse, according to our pre-questionnaire, the vast majority of students does not know where to seek help when confronted with scientific misconduct. In general, doctoral supervisors would likely come to mind as the first person a student would and should confide in when looking for help and advice. However, many supervisors feel poorly prepared for the tasks associated with a medical doctorate relationship (Can et al. 2016). When students are not provided with the supervision and guidance they need, this can, however, lead to serious consequences (Yahaghi et al. 2017): As research has for example shown, 20–30% of medical students in Germany do not complete their dissertation (Diez et al. 2000; Can et al. 2016). What is more, when students do not know who they can consult in addition to or instead of supervisors or colleagues, it has to be feared that they might not be able to recognize poor research behaviour and identify it as ethically problematic, or might do so, but do not know who to turn to, or might even copy ethically problematic patterns observed in others.

To enable students to comply to the principles of GSP, it seems thus crucial to make them not only aware of said rules, but also to familiarize them with the tasks, goals and current members of their university’s go-to authorities (ombudsperson). To tackle this challenge, we complemented the hypothetical case vignettes used during teaching with excerpts from guidelines out of scientific practice such as the German Research Foundations and the University’s statutes for safeguarding GSP (Ulm University 2009; German Research Foundation 2013). This approach served the goal to introduce students to the institutions and guidelines they can consult when fearing poor research behaviour in themselves or others, to make non-transparent processes transparent, and to familiarize them with the main ideas and processes of scientific self-control. The pre-post teaching comparison suggests the aptness of this approach, considering the statistically significant increase in participants’ expressed familiarity with the country’s main institutions and recommendations on GSP (Figs. 1, 2) and the office of ombudsperson as the university’s go-to authority for questions of GSP. This notion is additionally supported by the 30%-increase in students’—already initially confided—trust in this office (Fig. 3). Such an informed and trusting stance towards the institutions responsible for issues of GSP is the building stone for scholars’ ability to develop and apply solution strategies when witnessing or conducting poor research conduct, and thus to align their work with the principles of a scientific community founded on honesty and trust.

### Making GSP Training Compulsory

The vast part of the scientific research funding in Germany is invested in medicine, making it one of the largest research areas in the country (German Research Foundation 2019b). Society must be able to rely on scientists to handle the resources and tasks entrusted to them responsibly. Thorough and early preparation for a scientific career based on scientific integrity and honesty is a core component to create and maintain this trust. However, the integration and quality of scientific research training into German medical education has been criticized worldwide (Diez et al. 2000; German Research Foundation 2010; Loos et al. 2014; Editorial 2015; German Council of Science and Humanities 2015; Sennekamp et al. 2016; Epstein et al. 2018). One reason can be seen in our students’ expressed lack of working experience in a scientific research context e.g. as a research assistant, despite being already in the midst of their studies. This data corresponds to the country’s overall situation, as doctoral students in German medical schools did not receive any formal training in scientific work for a long time (Sponholz 2000, 2019, p. 64). This seems particularly problematic when considering the fact that the majority of German medical students earns a doctoral degree (“Dr. med.”), which makes this title the most frequently awarded doctoral degree in Germany (German Council of Science and Humanities 2011; Konsortium Bundesbericht Wissenschaftlicher Nachwuchs 2017, p. 92; Gillmann 2019). Another contributing factor is the lack of time usually invested in a doctoral project: In Germany, most students start working on a dissertation project during their undergraduate studies and thus while continuing to study full-time, which often goes to the expense of quality (Nature Editorial 2015; German Association of University Professors and Lecturers 2016). In light of these circumstances, our students’ expressed hesitation to pursue a career in sciences may come as no real surprise.

What is mostly highlighted in literature in this respect is the need to improve students’ knowledge and competences with regard to scientific writing, scientific methods, and statistical knowledge (Sennekamp et al. 2016; Epstein et al. 2018). Solid knowledge in all these areas is also vital for understanding the importance and implications of GSP. However, both the project’s pilot phase (Fuerholzer et al. 2019) and the data at hand suggest that students have limited abilities to identify and react to scientific misbehavior like plagiarism, which indicates that it is paramount to provide students not only with a scientific training in general but also with a solid training in scientific integrity (Fuerholzer et al. 2019). Fortunately, more and more German universities offer courses on GSP. Nevertheless, most research training programs are voluntary (Kuhnigk et al. 2010), which seems to be also true for GSP. In light of the experiences of our project, it seems however doubtful whether the task of qualifying students for scientific work can be adequately addressed when it is left to their decision to engage with GSP. The vast number of students for whom our course constituted their first contact with issues of GSP can be seen as proof of this risk, which is additionally stressed by the initially low level of knowledge yielded in the pre-teaching questionnaire. It is due to these critical circumstances that the course presented in our paper was implemented into the mandatory curriculum of all medical students at Ulm University. This decision is also understood as an answer to the recommendation of the German Research Foundation to impart the basics of GSP at the earliest possible point in academic teaching (German Research Foundation 2019a, pp. 9–10). Providing comprehensive training on the principles of GSP to all future scholars seems imperative when we want to ensure that the country’s support of, dependency on and trust in medical research can be adequately met. The high importance our students ascribed to GSP seems like a promising sign for their readiness to accept this challenge.

### Limitations and Strengths

Due to the rather short time span between pre and post evaluation, research was limited to cover short-term effects of GSP courses on students’ knowledge and attitude. Further studies on long-term outcomes are warranted. Also, comparative in-depth-analyses of the impact of heterogenous factors like students’ national or cultural origin, religious believes, educational background, sexuality etc. on issues of GSP seem necessary to design courses that are inclusive of all participants. Next to that, the paper at hand was strictly focussed on medical students; independent research is needed in how far the results presented in this paper can serve as a model for other health and life sciences like dentistry, nursing studies, pharmacy, molecular medicine, to name just a few. The goal of the questionnaire survey was to assess the knowledge and attitudes towards core concepts, institutions and guidelines on GSP of all students in the 3rd year of university. The results are thus based on a representative homogeneous sample group. In this context, the pre-post evaluation had its strength in giving profound insight into the background knowledge and attitudes of medical students in the midst of their academic education. To the best of our knowledge, this is the first study that considers the ethical necessity to implement mandatory courses on GSP into the curriculum of German medical students. A strength of the study are the high response rates at baseline which do not completely rule out possible selection bias but make it rather unlikely.

## Conclusion

Young scholars on the very beginning of their scientific career cannot command the practical and theoretical stock of knowledge and expertise which can only be gained by years of experience. Nevertheless, as soon as medical students embark on a dissertation project, they are obliged to follow the rules of GSP. Thus, it seems vital to provide all students with equal training on GSP, as it was the case in the project presented in this article. The pre-post-comparison revealed significant improvement in students’ skills to recognize poor research conduct in the given hypothetical scenarios and to identify it as ethically problematic; also, the post-questionnaire indicates students’ increased acquaintance with and trust in core insitutions and authorities on GSP. All of these are important presuppositions for the ability to apply theoretical knowledge on GSP into practice. Failure to inform students about these principles, institutions and guidelines of GSP can constitute itself a breach of the rules of GSP. However, unified regulations regarding the content, extent and format of courses on GSP in Germany are still needed. In light of the vast number of students involved in a doctoral research project while still at medical school, we highly recommend to integrate courses on GSP into the mandatory curriculum of all medical students already at an early stage of their training. In light of the experiences gained during the course of our project, we are convinced that students’ scientific integrity training needs to become as important as their medical one. At the same time, our data also suggests students’ willingness and ability to assume the challenge of pursuing a scientific career based on integrity and honesty.

## Figures and Tables

**Fig. 4:**
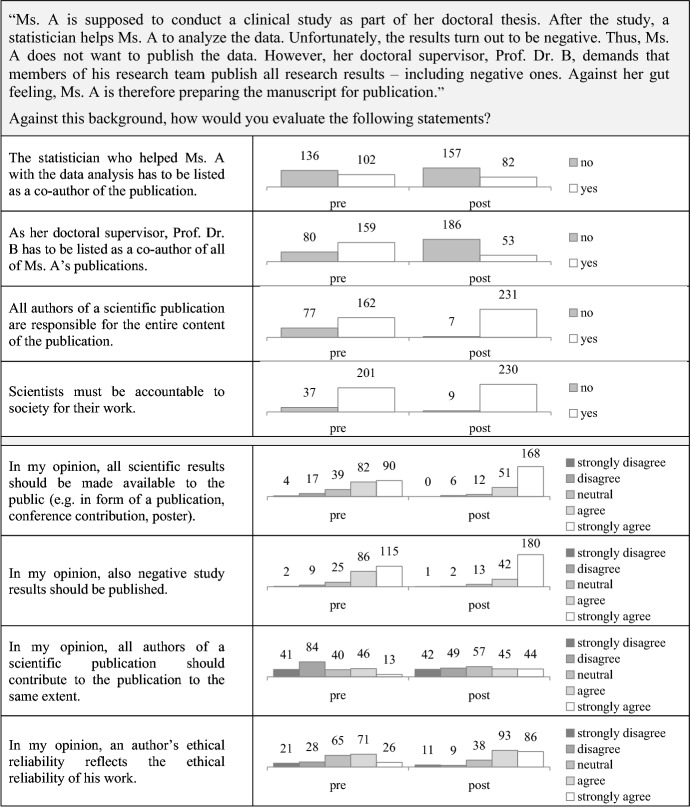
Hypothetical case vignette 1: Students’ knowledge and attitudes on GSP pre- versus post-teaching in absolute numbers (n = 239)

**Fig. 5:**
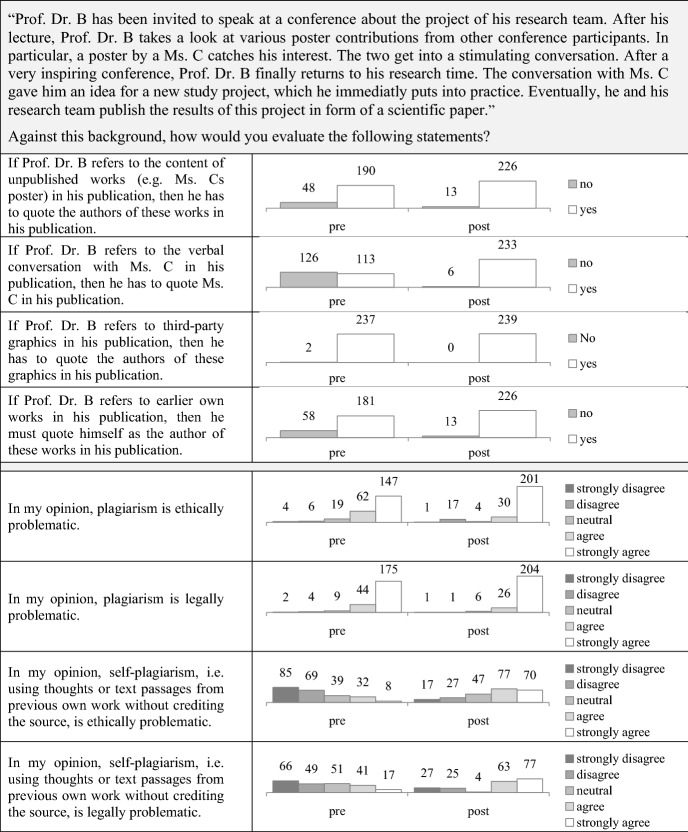
Hypothetical case vignette 2: Students’ knowledge and attitudes on GSP pre- versus post-teaching in absolute numbers (n = 239)

**Fig. 6:**
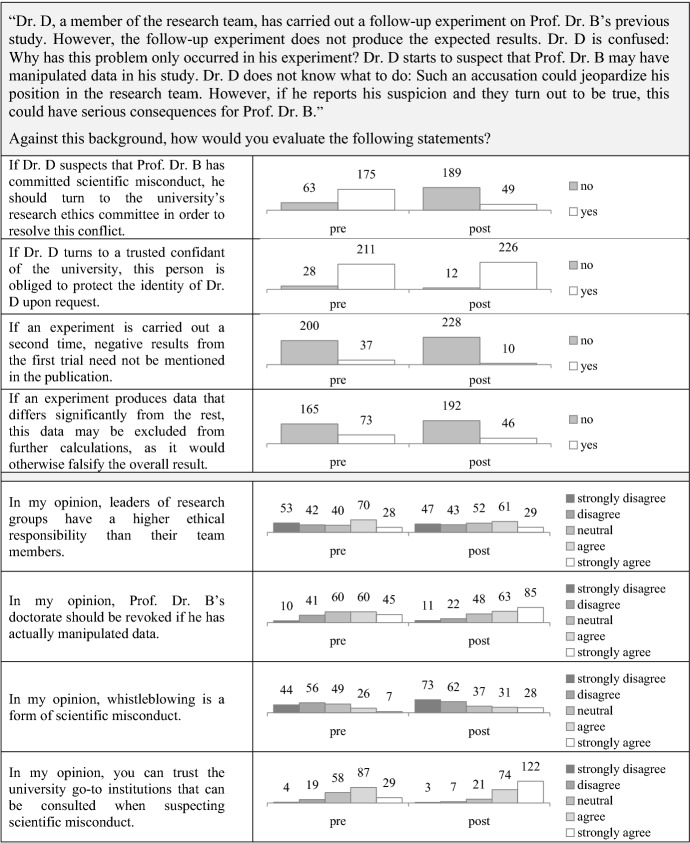
Hypothetical case vignette 3: Students’ knowledge and attitudes on GSP pre- versus post-teaching in absolute numbers (n = 239)

## Appendix: Students’ Knowledge and Attitudes on GSP Pre- Versus Post-teaching

See Figs. 4, 5, and 6.
